# Supplementary material for: Research on the economic effects of housing support expenditures under the perspective of consumption heterogeneity: Evidence from China
Source: PLoS One. 2024 Sep 12;19(9):e0306138. doi: 10.1371/journal.pone.0306138 (PMC11392229; doi:10.1371/journal.pone.0306138)
Supplement: Attachment — Submitted filename: Response 2.doc [file pone.0306138.s002.doc]

## Response to Reviewer 2

This study empirically investigates how government housing support spending affects urban families' overall and consumption-structured consumption using panel data at the province level.

The salient features of the current study are:

• The significance of the study is intelligibly highlighted

• The research problem is genuine and has regional and local appeal for welfare outcomes

• The research gap is well-identified and bridged

• The theoretical framework is constructed on simple but sound and pertinent theories of economics

• Operational definitions of the variables used in the model are very clear and their measurements (and units of measurement) are appropriate

• The results are logically interpreted and discussed in comparison with the previously conducted relevant studies.

**Response: Thank you, reviewers, for seeing merit in our work.**

Q1: The abstract is too long, especially, the part of the abstract presenting the study's findings. So, it must be shortened.

Q2: The last two sentences concluding the abstract are confusing and, hence, need to be clarified.

Response: Thank you for these insightful comments. We have revised the concerns you raised in the manuscript accordingly. Details of the revisions can be found in **Lines 9-21** of the paper, where we have reorganized and re-written it.

Q3: On page 4 (lines 161-162) clarify the sentence: “Beneficial increase in local residents' economic activity [26]”.

Response: Thank you for your careful review. We have reorganized and rewritten the literature review. Please see **Lines75-135** the revised manuscript for detailed revisions.

Q4: On page 6 (lines 242-253 ): use the punctuation and that of the sentence case appropriately.

Response: Thank you. We have reorganized and rewritten it. Please refer to section 2.2 ( Lines 137-171 ) of the text for detailed revisions.

Q5: On page 10 (lines 383-384): clarify the sentence: “This paper will be removed from the sample of the Shanghai Municipality, Tibet Autonomous 384 Region, as more data is missing.”

Response: Thank you for the suggestion. We have clarified this section. Please see **Lines 180-186** of the new paper for more details.

Q6: Justify, in tables 7, 8, and 9, for GMM estimation, why and how each explained variable is split into two.

Response: Tables 7-9 explore the consumption structure of urban residents. Separate bases, relevance to housing, necessity, and durability. This paper divides the structure of consumption expenditures into three aspects: housing-related consumption expenditures (variable Consume1 in Table 7), housing-unrelated consumption expenditures (variable Consume2 in Table 7); necessity consumption expenditures (variable Consume3 in Table 8), non-necessity consumption expenditures (variable Consume4 in Table 8); and consumption expenditures on durable goods (variable Consume5 in Table 9), and consumption expenditures on non-durable goods (variable Consume6 in Table 9).
